# Hyperspectral TERS Imaging Reveals Strain Heterogeneity in Individual Nanoplastic Particles

**DOI:** 10.1021/acs.nanolett.5c05003

**Published:** 2025-12-12

**Authors:** Anushree Dutta, Siiri Bienz, Naresh Kumar, Renato Zenobi

**Affiliations:** Department of Chemistry and Applied Biosciences, 27219 ETH Zurich, CH-8093 Zurich, Switzerland

**Keywords:** nanoplastic, TERS, hyperspectral imaging, nanoscale analysis, label-free measurements

## Abstract

Nanoplastics pose growing environmental and health risks, yet their label-free, nondestructive detection and characterization, especially at the single-particle level, remain challenging. Here, we deploy AFM-based tip-enhanced Raman spectroscopy (AFM-TERS) to chemically characterize individual polystyrene (PS) nanoplastic particles via hyperspectral imaging under ambient conditions. TERS spectra from nanoparticles as small as 32 nm establish reliable single-particle sensitivity beyond the optical diffraction limit. Furthermore, hyperspectral TERS maps reveal pronounced intraparticle heterogeneity, reflected spatially as varying red-/blue-shifts of PS marker bands with broad frequency distributions, without any systematic dependence on particle size. Correlative AFM phase imaging exposes nanoscale variations in local stiffness indicating strain heterogeneity as the origin of the spectral shifts. These results demonstrate that AFM-TERS enables single-particle mapping of intraparticle heterogeneity in nanoplastics. This offers new possibilities to identify nanoplastics with molecular specificity and monitor chemical transformations at the single-particle level within complex biological and environmental matrices.

## Graphical abstract

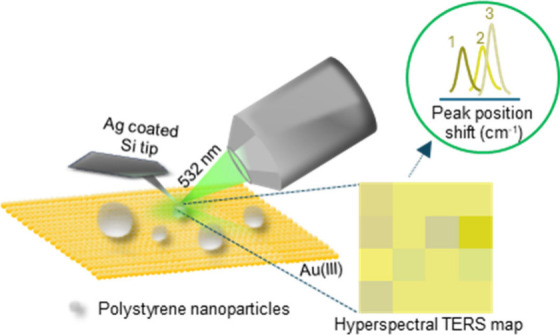

Plastic pollution has emerged as a major global challenge due to the vast scale of plastic production and consumption, coupled with inadequate waste management.^1^ Environmental weathering fragments bulk plastics into micro- and nanoplastics, raising pressing ecological and health concerns. Nanoplastics are polymer particles that are generally distinguished from microplastics by size, with nanoplastics commonly defined as having 1–100 nm dimensions and microplastics with sizes of 1 μm–1 mm.^2–4^ Some reports extend the upper nanoplastic size toward 100–1000 nm, although this lies outside the formal nanoscale definition. From a health perspective, nanoplastics are especially hazardous because their small dimensions confer high reactivity and enable them to cross biological barriers, with documented accumulation in tissues and interactions with cellular organelles that induce oxidative stress and other impairments of cell function.^5–8^ Owing to their high surface-to-volume ratio, nanoparticles are strongly governed by interfacial effects, with altered physicomechanical properties compared to their bulk counterparts.^9^ For example, in confined polymer systems, polymer chain conformations deviate from those in the equilibrium melt, generating spatial variations and nanoscale heterogeneity.^9,10^ Similar effects are expected in nanoplastics, where local differences in chain packing can dictate the mechanical stability, chemical reactivity, and interactions with biological interfaces. Such nanoscale heterogeneity, while critical for determining the fate and potential toxicity of nanoplastics, remains poorly understood. Although conventional characterization techniques like atomic force microscopy (AFM), transmission electron microscopy, and scanning electron microscopy can unveil nanoscale heterogeneity based on surface morphology at the single-particle level, they provide limited chemical information.^11,12^ This gap underscores the urgent need for label-free, nondestructive analytical approaches that offer nanoscale spatial resolution to probe, chemically characterize, and image individual nanoplastics under ambient conditions, which could serve as a potential tool in analyzing nanoplastics in complex environmental and biological samples in the future.

To identify and quantify plastic particles in complex matrices, pyrolysis–gas chromatography–mass spectrometry is a widely used method; however, it is destructive, is prone to matrix interferences, and provides no information on particle size, shape, or morphology.^13^ Fluorescence-based approaches require labeling, which necessitates additional sample preparation steps and can alter nanoplastic surface chemistry.^14,15^ Bulk microplastics can be chemically identified by Fourier-transform infrared spectroscopy or confocal Raman spectroscopy;^16,17^ however, these techniques face limitations at trace concentrations or at the single-nanoparticle level, due to limited sensitivity and diffraction-limited spatial resolution.^13,14,18,19^ SERS has enabled the detection of individual polymeric particles of less than 100 nm using custom-engineered substrates.^20,21^ Yet it relies on complex sample and substrate preparation and further fails to reach a spatial resolution below the diffraction limit.^20,22,23^ Hyperspectral SRS with automated classification achieves high-throughput single-particle chemical imaging,^24^ but remains diffraction-limited and cannot resolve subparticle heterogeneity. AFM-IR has detected polymeric particles, such as polylactic acid, with diameters below 200 nm in cells; however, probing <100 nm nanoplastics remains challenging.^24,25^

Collectively, existing methods involve trade-offs among chemical sensitivity, molecular specificity, and nanoscale spatial resolution, leaving most analyses confined to ensemble measurements rather than label-free and nondestructive single-particle characterization. Here, we address this gap by employing tip-enhanced Raman spectroscopy (TERS) to probe individual PS particles and evaluate their surface chemical heterogeneity through hyperspectral Raman imaging under ambient conditions. TERS couples the nanometer precision of scanning probe microscopy with the molecular specificity of Raman spectroscopy, leveraging localized surface plasmon resonances at a noble metal tip to confine and amplify the electromagnetic near field at the sample, thereby enhancing both sensitivity and spatial resolution for hyperspectral mapping.^26,27^ TERS has been widely applied in a range of research areas, including heterogeneous catalysis,^28–30^ biomembranes,^31–33^ single-molecule studies,^34–37^ 2D materials,^38–40^ etc.; however, its use for nanoplastic research has remained underexplored. An additional challenge lies in intraparticle (subparticle level) imaging of nanoplastics to probe nanoscale heterogeneity, which governs their physicochemical behavior. Although TERS has been applied to resin-embedded or sectioned polymer nanoparticles, probing of nanoscale surface heterogeneity through hyperspectral TERS imaging at the single-particle level has hitherto not been achieved.^41^

Here, we probe polystyrene (PS) particles as a model nanoplastic system under ambient, label-free, and nondestructive conditions to demonstrate the effectiveness of TERS for nanoplastic research. TERS spectra successfully measured from individual PS nanoparticles down to 32 nm establish reliable single-particle sensitivity. Subsequent hyperspectral TERS mapping revealed intraparticle heterogeneity, evident by spatially varying red- and blue-shifts of the PS Raman marker bands. These frequency shifts exhibit a broad distribution without a systematic dependence on particle size; they were also found to vary across the nanoparticle surface. Complementary AFM phase imaging reveals a nanoscale variation in local stiffness indicating strain heterogeneity across the nanoparticle surface as a plausible origin of the spectral shifts. Together, these results demonstrate that AFM-TERS can chemically image individual nanoplastics while reporting on their nanoscale structural variability, providing a foundation for future studies of nanoplastics in complex environmental and biological matrices.

Figure 1 shows a schematic representation of the reflection mode AFM-TERS setup used in this study. A representative SEM image of a Ag-coated AFM tip used for the TERS measurements in this study is shown in the inset. Figure S1 shows the reference confocal Raman spectrum of the PS powder. PS exhibits characteristic Raman vibrational modes at 621 cm^–1^ (phenyl ring deformation mode), 794 cm^–1^ (C–H out of plane deformation), 1000 cm^–1^ (phenyl ring breathing), 1030 cm^–1^ (C–H deformation in-plane), 1153 cm^–1^ (ring C–C–H bending vibration), 1601 cm^–1^ (phenyl ring skeletal (C=C) stretching), 2904 cm^–1^ (aliphatic C–H stretching), and 3057 cm^–1^ (phenyl C–H stretching mode).^42–44^

**Figure 1:**
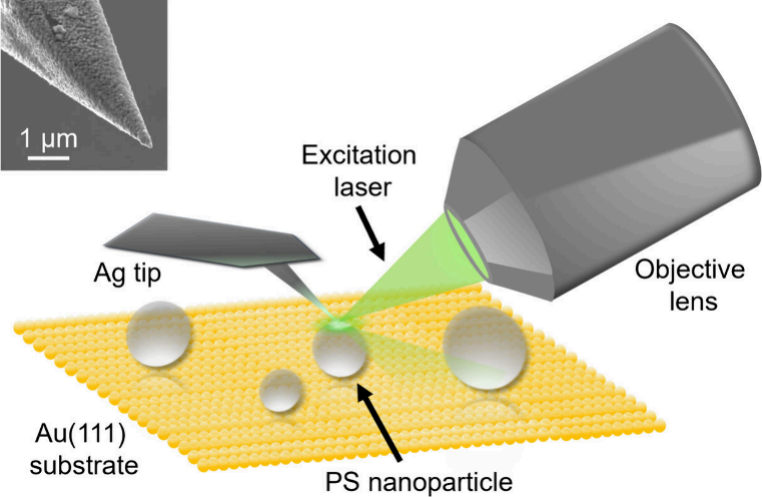
Schematic illustration of the side-illumination AFM-TERS setup employed for the hyperspectral imaging of single PS nanoparticles. The inset displays an SEM image of a representative Ag-coated TERS probe used in this study.

For TERS measurements, a colloidal dispersion of mixed-size PS particles was drop-cast onto a Au(111) surface and dried overnight. AFM topography images of the deposited PS particles are shown in Figures 2a–c, with the corresponding height profiles presented in Figures 2d–f. TERS spectra acquired from individual PS particles with diameters of 32, 73, 174, 176, 428, and 434 nm are displayed in Figures 2g–i. Distinct vibrational features characteristic of PS are observed, including sharp peaks at 1000–1006 cm^–1^, 3060–3061 cm^–1^, and ∼1601 cm^–1^ corresponding to the phenyl ring breathing (ν_RB_), phenyl C–H stretching (ν_C–H(aro)_), and phenyl C=C stretching modes, respectively, matching well with the reference PS spectrum in Figure S1.^42^ Control experiments under tip-in and tip-away conditions (Figure S2) confirm that Raman bands are detected only when the TERS tip is engaged, demonstrating the single-particle sensitivity of TERS. In contrast, far-field Raman spectra (tip-away) show no detectable signals, underscoring the inability of conventional Raman spectroscopy to probe individual nanoplastics. The peaks observed in the confocal Raman and TERS spectra of PS particles are summarized in Table S1.

**Figure 2:**
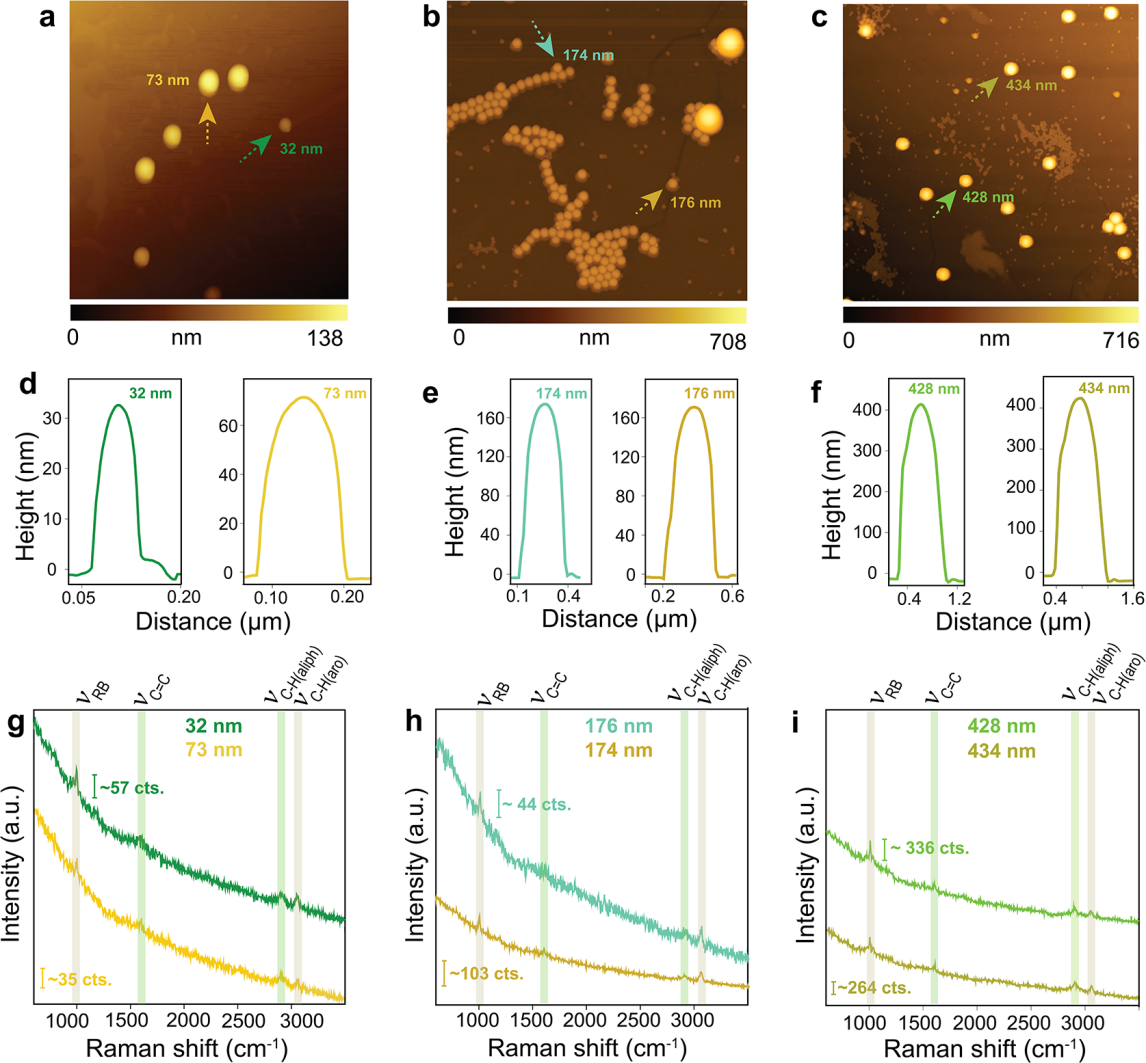
AFM topography images of PS particles with different sizes from samples with a multimodal size distribution (see Supporting Information for details): (a) 32 and 73 nm, (b) 174 and 176 nm, and (c) 428 and 434 nm. The corresponding height profiles of the PS particles are shown in panels d–f. TERS spectra acquired from the individual PS particles highlighted in panels a–c: (g) 32 and 73 nm, (h) 174 and 176 nm, and (i) 428 and 434 nm. Integration time for each spectrum was 3 s.

Polymers inherently exhibit nonuniformities in composition,^45,46^ crystallinity,^47,48^ molecular conformation,^34^ morphology,^45,49^ and strain, which can induce spatial heterogeneity at the nanoscale.^50,51^ For PS, variations in the local environment, molecular packing, and structural geometry are known to directly influence the aromatic ring breathing vibrational mode.^42,47,52^ For example, atactic PS, under shock compression, showed a blue-shift of the phenyl ring breathing mode by 5 cm^–1^ and an increase in its line-width.^53,54^ Therefore, the aromatic ring breathing mode of PS at ∼1000 cm^–1^, is a particularly reliable spectral marker to probe nanoscale structural, conformational, and mechanical characteristics.^42,54,55^

We performed statistical analysis of the ring breathing mode in the TERS spectra measured from 52 individual PS particles, which is presented in Figure S3. Notably, the vibrational frequency of the ring breathing mode showed substantial particle-to-particle variability, with broad and overlapping error bars across different size groups (30–80 nm, 100–200 nm, and 250–520 nm). These results indicate that frequency shifts of the ring breathing mode are random and independent of particle size, likely reflecting heterogeneities in local strain, conformation, or molecular packing at the single-particle level.

We next performed hyperspectral TERS mapping at the single-particle level. To mitigate positional drift, which was prevalent for PS particles < 200 nm deposited noncovalently on Au(111), we focused on larger PS particles (>400 nm). Particles with diameters of 512, 421, and 427 nm shown in Figures 3a–c­(corresponding height profiles in Figure S4) were selected for analysis.Figures 3. The corresponding hyperspectral maps of the intensity and position of the aromatic ring breathing mode are presented in Figures 3e–g and Figures 3i–k, respectively. For comparison, the AFM topography image and TERS intensity and peak position maps of a PS thin film are also presented in Figures 3d, h, and l, respectively.

**Figure 3:**
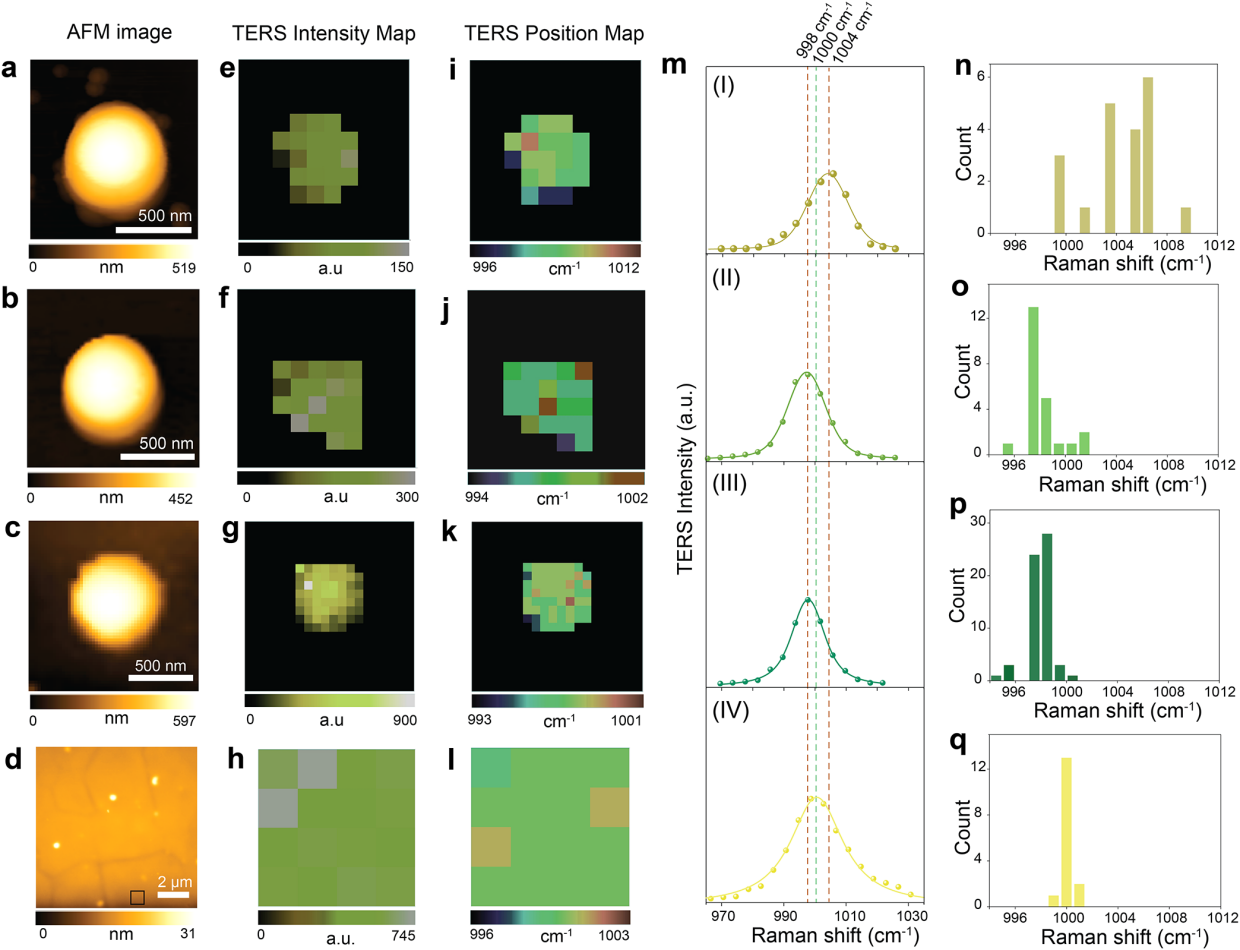
AFM topography images of individual PS particles with diameters of (a) 512, (b) 421, and (c) 427 nm. Hyperspectral TERS maps generated from (e–g) the intensity and from (i–k) the peak position of the ring breathing mode (obtained by peak fitting using a Voigt function) of the particles shown in panels a–c, acquired with step sizes of 121 nm (e), 90 nm (f), and 60 nm (g). Threshold values of 14, 33, and 22 counts (equal to the spectral noise) were applied to determine the presence of Raman signal in panels e–g and i–k, respectively; only pixels exceeding these thresholds were considered valid. The pixels showing no PS TERS signals were assigned a zero intensity value. (d) AFM topography of a PS thin film on a Au(111) surface and the corresponding hyperspectral TERS (h) intensity and (l) position maps generated from the ring breathing mode. (m) Average spectra (I–IV) extracted from the TERS maps in panels e–h. Histograms of the aromatic ring breathing mode peak positions extracted from individual pixels of the hyperspectral TERS maps of PS particles (i–l) with diameters (a) 512 nm, (b) 421 nm, and (c) 427 nm and of (d) a PS thin film, shown in n–q, respectively. The corresponding mean ± standard deviation of the peak position for each histogram plot shown in panels n–q are 1004 ± 2.7 cm^–1^, 998 ± 1.4 cm^–1^, 998 ± 0.9 cm^–1^, and 1000 ± 0.4 cm^–1^, respectively.

The waterfall plots of all spectra (spanning 660–3300 cm^–1^) drawn from the hyperspectral TERS maps corresponding to PS particles and the thin film shown in Figures 3a–c and d, respectively, are presented in Figure S6, where the characteristic Raman bands of PS are clearly visible. Further details of the spectral analysis are provided in the Supporting Information (Experimental Details section). Average spectra for each nanoparticle, obtained by integrating over all pixels in the maps in panels e–g, are shown in Figure 3m­(I–III). These results demonstrate the capability of hyperspectral TERS mapping for spatially resolved chemical analysis of individual nanoplastic particles. This is particularly relevant for probing nanoplastic at the single-particle level, where nanoscale heterogeneity in structure and composition is the most critical and less explored.

The average TERS spectra in Figure 3m reveal distinct frequency shifts in the aromatic ring breathing mode of PS particles compared with the PS thin film. Specifically, shifts of +4 cm^–1^ (I, 1004 cm^–1^) and −2 cm^–1^ (II and III, 998 cm^–1^) are observed for the 512, 421, and 427 nm particles, whereas the thin-film spectrum (IV) exhibits no shift and remains centered at 1000 cm^–1^. To further investigate this variability, peak positions were extracted from each pixel of the TERS maps (Figures 3i–l) and plotted as histograms (Figure 3n–q). The 512 nm nanoparticle (Figure S4a) shows a broad distribution with the most frequent peak at ∼1006 cm^–1^ (Figure 3n), while the 421 nm nanoparticle (Figure S4b) and 427 nm (Figure S4c) particles also exhibit wide distributions but with dominant peaks at 997 cm^–1^and 998 cm^–1^ (Figures 3o and p). These modal values are consistent with the peak positions in the corresponding average spectra (Figure 3m). In contrast, the PS thin film displays a narrower distribution (Figure 3q), with the most frequent peak position at 1000 cm^–1^. Hyperspectral TERS imaging reveals that PS particles exhibit a broader spatial distribution of vibrational frequencies compared to the thin film. Since strain-induced red and blue shifts in vibrational bands are well documented for polymer and inorganic thin films,^54,56^ the spatial variation in vibrational frequencies observed in hyperspectral TERS maps indicates a heterogeneity of local strain at the single-particle scale. This heterogeneity likely arises from local structural and orientational variations of PS molecules across the nanoparticle surface and is particularly relevant for nanoplastics, since such variability may govern their reactivity, degradation behavior, and interactions in complex environmental systems.

Since the PS dispersion used in this study consisted solely of PS particles (with only trace additives) obtained from a commercial source, the polymer is expected to be compositionally and structurally homogeneous (atactic).^53^ To further probe the observed surface heterogeneity, we performed high-resolution AFM imaging of three PS particles with diameters of 72, 186, and 462 nm and PS thin film and measured their topography (Figure S5) and phase images (Figure 4). While AFM topography provides height information, phase imaging offers contrast related to variations in local mechanical properties, such as stiffness, or local surface chemistry, etc.^11,57,58^ For example, surface heterogeneity in quaternary ammonium chloride polystyrene latex particles arising from the separation of ion-rich and ion-poor surface domains was clearly distinguished by AFM phase contrast imaging.^11^ Because the phase shift can be influenced by several experimental parameters, including the set point, drive amplitude, cantilever properties, environmental conditions, etc., the data provide qualitative rather than quantitative information. For a flat, uniform sample, minimal contrast in the phase image is expected, aside from slight variations at edges due to changes in tip–sample interactions.

**Figure 4:**
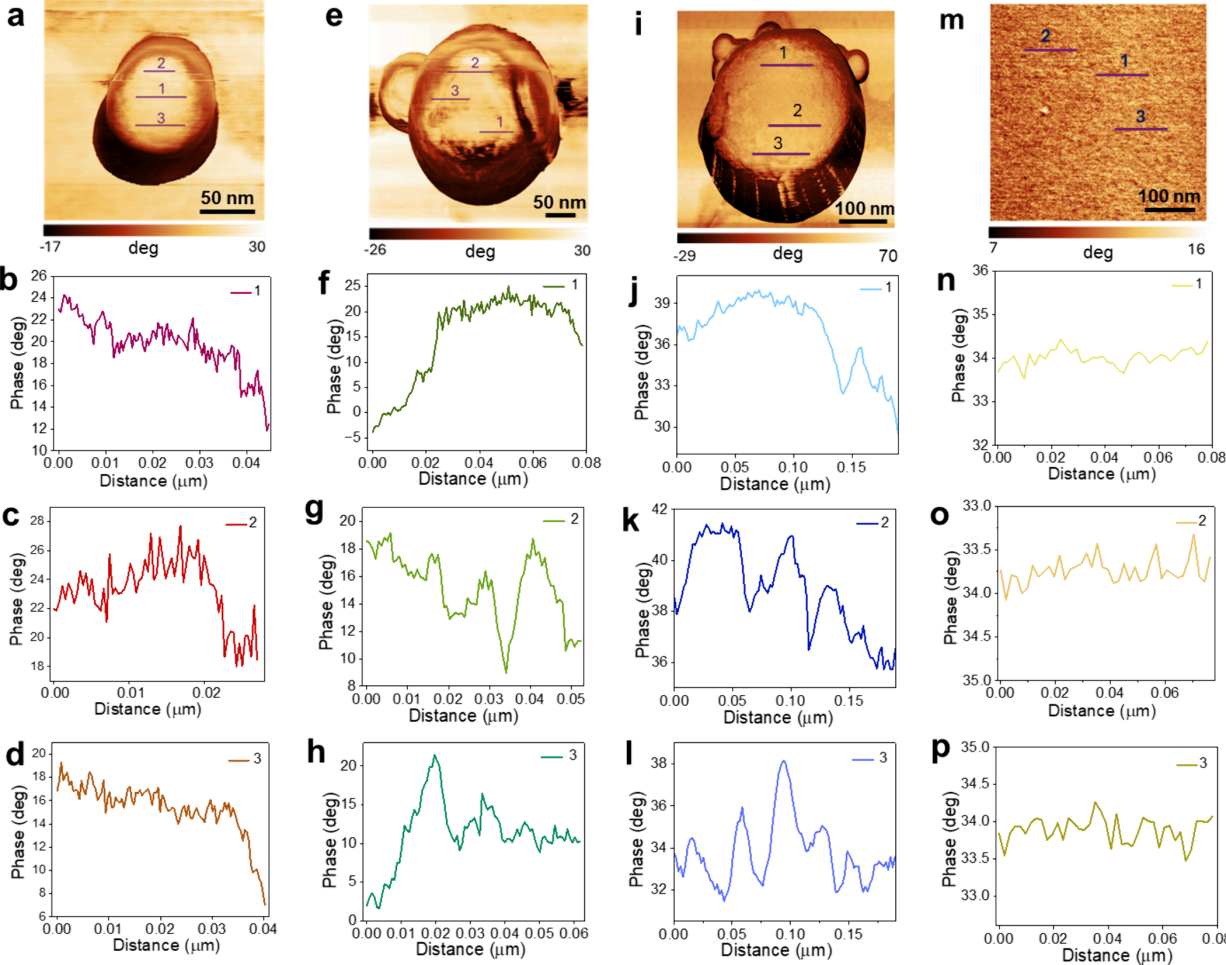
AFM phase images of PS particles with diameters of (a) 72, (e) 186, and (i) 462 nm, corresponding to the particles shown in Figures S5a–c. Cross-sectional traces marked as 1, 2, and 3 in panels a, e, and i are presented in panels b–d, f–h, and j–l, respectively. For comparison, the AFM phase image of a PS thin film is shown in panel m, and cross-sectional traces marked as 1, 2, and 3 are presented in panels n–p. Unlike the phase image contrast observed in panels b–d, f–h, and j–l, the thin-film cross-sectional traces did not reflect a striking stiffness contrast.

AFM phase images of the 72, 186, and 462 nm PS particles (Figures 4a, e, and i) reveal notable contrasts across the particle surfaces. We analyzed phase profiles extracted from the regions away from the edges of the particles. Cross-sectional traces extracted from multiple positions on each particle (Figures 4b–d, f–h, j–l) show relative phase lags that vary from region to region. Brighter regions with more positive phase shifts correspond to areas of reduced stiffness, indicating greater energy dissipation during tip–sample interaction, whereas regions with less positive (or negative) phase shifts correspond to stiffer domains with reduced damping.^58^ In all three PS particles, sharp dips in the phase angle are evident, marking localized stiff regions. In contrast, the phase image of the PS thin film (Figure 4m) displays no such striking contrast in stiffness, as evident from the cross-sectional traces shown in Figure 4, panels n–p. Since local stiffness variations reflect differences in the elastic modulus, which is correlated with local strain, the nanoscale stiffness variation observed in the phase images indicates the presence of differential strain across the surface of PS particles.^59^ This is likely arising from nonuniform molecular packing, density, or crowding of PS chains at the particle surface, which is often observed in confined polymer systems.^9,42,50,51,54^ Such molecular effects can impose tensile (red-shift) or compressive (blue-shift) strain, leading to frequency shifts in vibrational modes,^53,60^ consistent with those observed for the aromatic ring breathing mode in Figure 3. These results suggest that the frequency shifts detected by TERS could arise from nanoscale variations in stiffness across the nanoparticle surface, underscoring the local strain heterogeneity of PS particles.

In this work, we demonstrated the utility of AFM-TERS as a powerful nanoanalytical tool for the characterization of individual nanoplastic particles with single-particle sensitivity down to 32 nm. Importantly, the measurements were performed in a label-free manner under ambient conditions and are nondestructive by nature, thereby preserving the intrinsic chemical and physical properties of the particles, which is an essential requirement for accurate nanoplastic analysis. We demonstrated the intrinsic local strain heterogeneity that exists across the surface of individual nanoplastics by topographical, phase, and hyperspectral TERS imaging, information that is inaccessible by conventional methods. This capability is particularly significant for characterizing commercially derived nanoplastics commonly employed in kinetic, drug delivery, and biosensing studies, where particle-to-particle variability may influence performance and behavior. AFM-TERS imaging enables the correlation of nanoscale chemical fingerprints with local structural heterogeneity, providing unique insight into nanoscale strain effects that exist at the single-particle level. The precision provided by AFM-TERS to resolve the nanoscale properties of individual nanoplastics further opens up the possibilities of probing plastic particles in complex matrices and tracking their chemical transformations in environmentally and biologically relevant settings.

## Supplementary Material



## Data Availability

The original data used in this publication will be made available in a curated data archive at ETH Zurich (https://www.researchcollection.ethz.ch) under the DOI 10.3929/ethz-c-000788482.

## Notes

Experimental details including materials and methods, confocal Raman spectra of PS, assignment of confocal Raman and TERS peak, AFM topography images of PS particles (diameters 73, 175, and 314 nm) and corresponding single-particle TERS spectra, statistical distribution of the aromatic ring breathing vibrational band (52 PS particles), height profiles of 512, 421, and 427 nm PS particles, AFM topography images of PS particles (72, 186, and 462 nm) and corresponding height profiles, waterfall plots of PS particles (512, 421, and, 427 nm) and PS thin film (PDF)
