# Supplementary material for: Hyperspectral TERS Imaging Reveals Strain Heterogeneity in Individual Nanoplastic Particles
Source: Nano Lett. 2025 Dec 12;25(51):17806–13. doi: 10.1021/acs.nanolett.5c05003 (PMC12751103; doi:10.1021/acs.nanolett.5c05003)
Supplement: Supplementary file 1 [file nl5c05003_si_001.pdf]

Supplementary Information

Hyperspectral TERS Imaging Reveals Strain Heterogeneity in Individual Nanoplastic Particles

Anushree Dutta, Siiri Bienz, Naresh Kumar*, and Renato Zenobi*

Department of Chemistry and Applied Biosciences, ETH Zurich, CH-8093 Zurich, Switzerland

*Email: naresh.kumar@org.chem.ethz.ch, zenobi@org.chem.ethz.ch

Experimental details

Materials. Polystyrene (PS) nanoparticle standards with a multimodal size distribution (nominal diameters: 81, 203, and 508 nm) were obtained as an aqueous dispersion (Duke Standards, Thermo Scientific). ACCESS-FM silicon AFM probes were purchased from AppNano (USA). High-purity silver wire (AG5501, 99.99%) was obtained from Advent Research Materials (UK). Au(111)-coated mica substrates were sourced from Georg Albert PVD (Germany). All materials were used as received unless otherwise noted.

Sample preparation. A 10 μL aliquot of PS nanoparticle dispersion (Duke Standards) was diluted in 400 μL of Milli-Q water and mixed thoroughly. The suspension was purified by centrifugation at 10,000 rpm for 10 min, and the resulting pellet was redispersed in 410 μL of Milli-Q water, followed by sonication for 5 min. For single-particle measurements, 30 μL of this dispersion was drop-cast onto an Au(111)-coated mica substrate and dried overnight under ambient conditions. For thin-film preparation, PS (molecular weight ~100,000; GPC standard, Sigma-Aldrich) was dissolved in chloroform to a concentration of 20 mg/mL. The solution was spin-coated onto a clean Au(111)-coated mica substrate at 2500 rpm for 5 min under a nitrogen atmosphere. Samples were stored in a nitrogen environment before measurements to ensure stability.

AFM measurements. AFM topography and phase images were acquired in tapping mode under ambient conditions using Ag-coated TERS probes. Image processing was performed, and particle height profiles were extracted with *Gwyddion* software.

TERS probe preparation. Si AFM probes were first oxidised in a furnace (Carbolite Gero, UK) at 1000 °C for 23 h to reduce the surface refractive index, followed by UV–ozone cleaning (Ossila, UK) for 1 h. The probes were subsequently coated with Ag using a built-in thermal evaporation system housed in a nitrogen glovebox (MBraun, Germany). A standardised protocol was employed to deposit a 120 nm Ag layer at a rate of 0.05 nm/s under a base pressure of 1×10^{-7} mbar. The Ag-coated probes were stored in a nitrogen glovebox (<0.1 ppm O_2 and H_2O) before use to prevent contamination.

TERS measurements. TERS experiments were conducted using an integrated AFM–Raman system comprising a Raman spectrometer (LabRam Soleil, HORIBA Scientific, France) coupled to an AFM (HORIBA Scientific, France) in a side-illumination geometry. The excitation laser was focused onto the probe apex using a 100×, 0.7 NA objective at an incident angle of 60° relative to the surface normal. Before each TERS experiment, an AFM topography image of the region of interest was acquired. Single-point spectra were then recorded by positioning the TERS probe near the centre of individual nanoparticles of different sizes. Hyperspectral TERS maps were collected using a laser power of 3.2 mW with an acquisition time of 1 s per spectrum in "SpecTop mode," wherein the AFM alternates between tapping and contact modes: spectra are acquired in contact mode while pixel-to-pixel transitions occur in tapping mode.

TERS data were processed using LabSpec software (v. 6.7.1.10). Raman maps were generated from the average intensity in the 965–1035 cm^{-1} range after baseline subtraction. Spectra from each pixel were individually smoothed (Savitzky–Golay, factor 2) and the peak position is determined by peak fitting with a Voigt function within the 965–1035 cm^{-1} spectral window. For TERS maps, manual data inspection was performed: pixels with no detectable PS TERS signals or with intensities below the noise threshold were assigned zero intensity. The waterfall plot of spectra extracted from each pixel of the respective TERS map of PS nanoparticle are generated using Origin software.

Spectral Resolution calculation: The spectral resolution of the Raman spectrometer was determined using a Ne calibration lamp by measuring the full width at half maximum (FWHM) of the Ne line at 585.25 nm via Voigt function fitting. This yielded a spectral resolution of 9.4 cm^{-1} or 0.32 nm. Note that despite the large spectral resolution of Raman spectrometer, subtle changes in the Raman peak positions can still be discerned via careful peak fitting.

SEM Measurements. SEM imaging of Ag-coated tips was performed on a Zeiss Auriga Compact Crossbeam system equipped with a Gemini field-emission electron column and an InLens secondary electron detector, operated at an accelerating voltage of 5 kV.

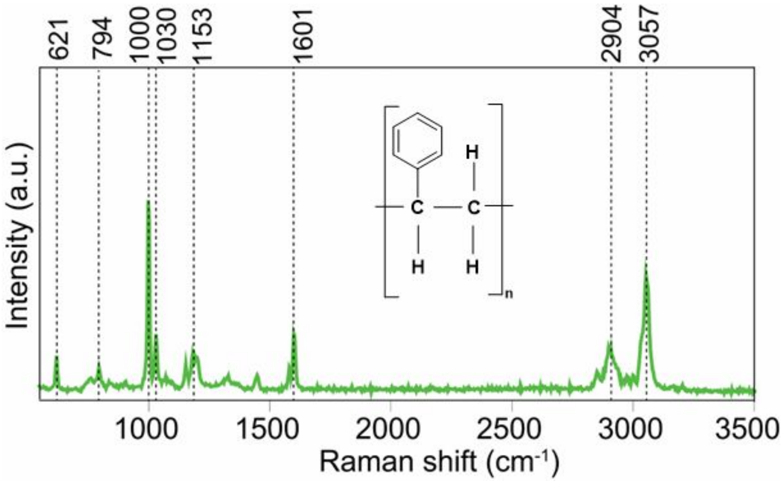

Figure S1. Confocal Raman spectrum of bulk PS powder. Excitation laser: 532 nm laser. Integration time of the spectrum was 5 s.

Table S1. Table summarising the peaks observed in the confocal Raman and TERS spectra of PS particles.

Band Assignment	Peak position	
	Confocal Raman (cm^{-1})	TERS (cm^{-1})
Phenyl ring deformation	621	
C-H out-of-plane deformation	794	
Phenyl ring breathing, ν_{RB}	1000	1000-1006
C-H deformation in-plane	1030	
C-C-H ring bending vibration	1153	
C=C stretching, phenyl ring	1601	1601
Aliphatic C-H stretching, $\nu_{C\text{-}H}$	2904	
phenyl ring C-H stretching, $\nu_{C\text{-}H}$	3057	3060–3061

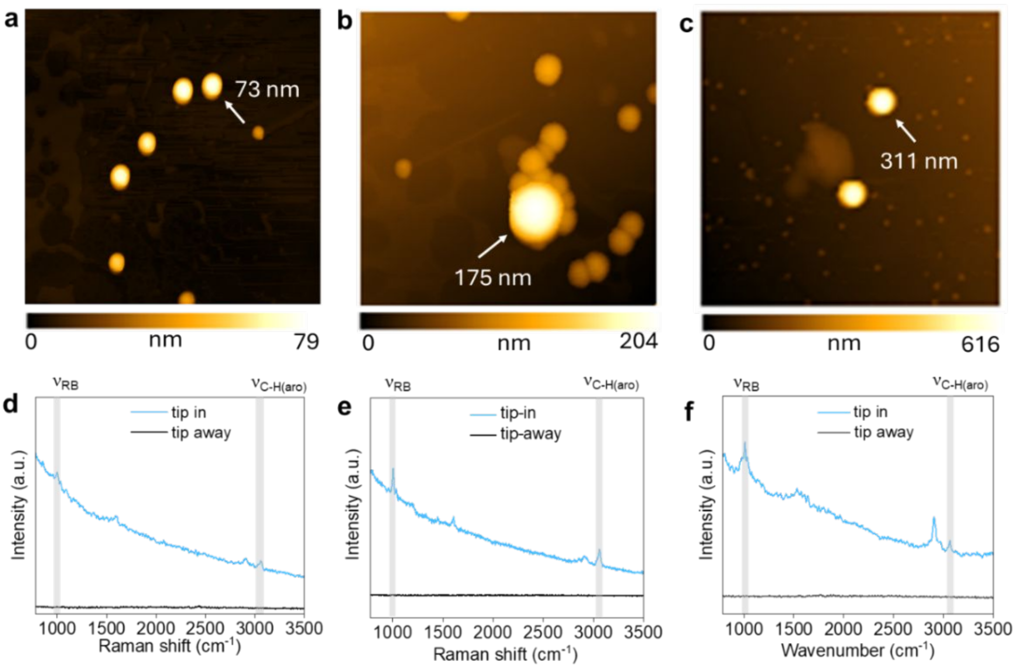

Figure S2. AFM topography images of PS particles with diameters of (a) 73 nm, (b) 175 nm, and (c) 314 nm, highlighted by arrows. The corresponding single-particle TERS spectra (blue) of the highlighted particles are shown in (d–f), together with the far-field (tip-away) spectra (bottom spectrum (black) in each Panel). Acquisition time for each spectrum was 2 s. Raman bands are observed only when the TERS tip is in contact with an individual particle, demonstrating the single-particle sensitivity of TERS. In contrast, far-field Raman spectra lack detectable signals, underscoring the inability of conventional Raman spectroscopy to probe individual nanoplastic particles at this scale.

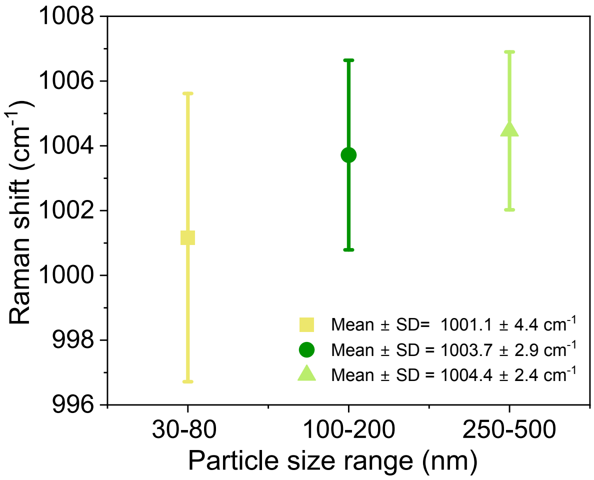

Figure S3. Statistical distribution of the peak position of the aromatic ring breathing mode of a total of 52 PS particles, grouped by size ranges of 30–80 nm (N= 18 particles), 100–200 nm (N= 8 particles), and 250–520 nm (N= 26 PS particles). Data are presented as mean ± standard deviation (SD). Substantial particle-to-particle variability is evident from the broad and overlapping error bars, indicating that the observed shifts in the ring breathing mode peak position are random and independent of particle size.

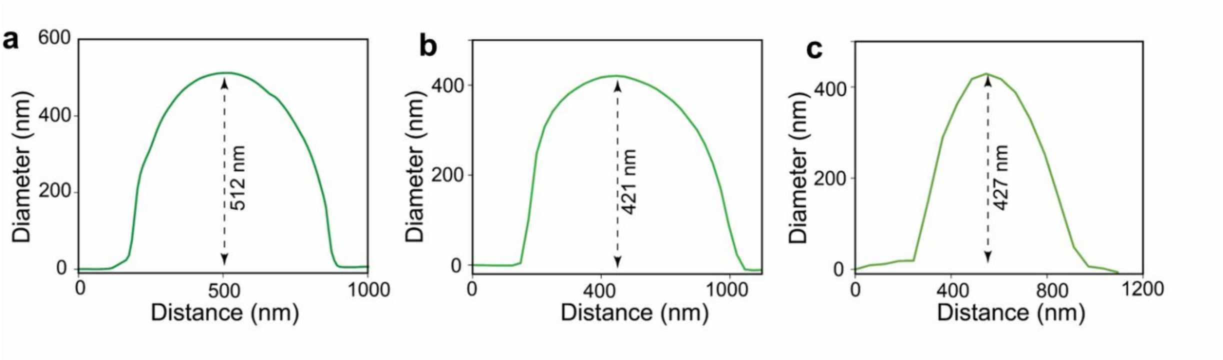

Figure S4. Height profiles of the (a) 512 nm, (b) 421 nm, and (c) 427 nm PS particles depicted in Figures 3a-c, respectively.

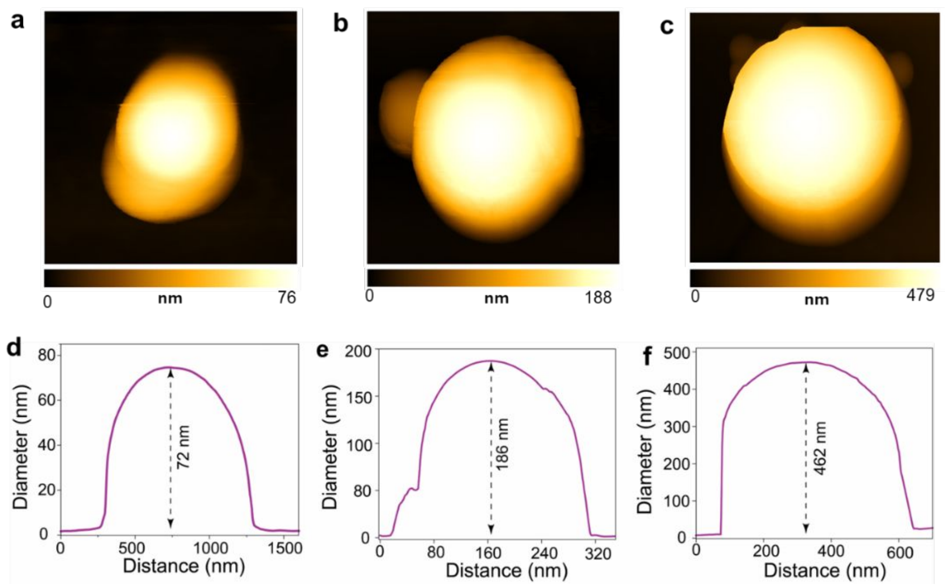

Figure S5. AFM topography images of PS particles with diameters of (a) 72 nm, (b) 186 nm, and (c) 462 nm, with the corresponding height profiles shown in panels d–f, respectively.

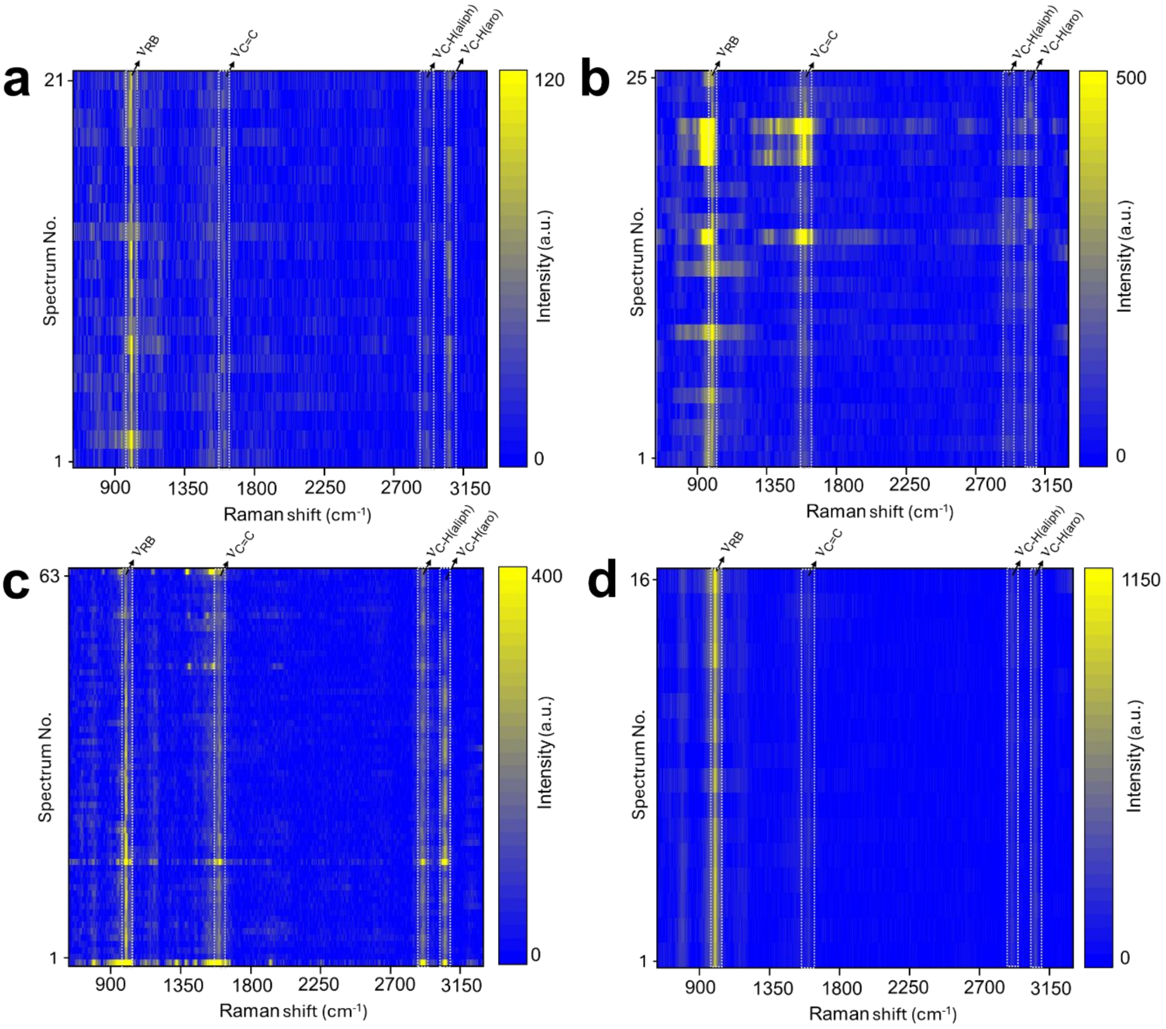

Figure S6. Waterfall plots of the TERS spectra of PS particles and thin-film drawn from the hyperspectral TERS maps shown in (a-c) Figures 3a-c, (d) and Figure 3d, respectively. Only the TERS spectra showing PS signals are included in the waterfall plots. The characteristic PS Raman bands are labelled.
